# Clustering change patterns among learners of an online Recovery College in Quebec

**DOI:** 10.3389/fpsyt.2025.1534349

**Published:** 2025-05-27

**Authors:** Filippo Rapisarda, Catherine Briand, Catherine Vallée, Brigitte Vachon, Galaad Lefay

**Affiliations:** ^1^ Département d’ergothérapie, Université du Québec à Trois-Rivières, Trois-Rivières, QC, Canada; ^2^ Centre d’études sur la réadaptation, le rétablissement et l’insertion sociale, Centre de Recherche de l’Institut Universitaire en Santé Mentale de Montréal, Montréal, QC, Canada; ^3^ School of Rehabilitation Sciences, Université Laval, Québec, QC, Canada; ^4^ School of Rehabilitation, University of Montreal, Montréal, QC, Canada

**Keywords:** Recovery College, outcome, cluster analysis, empowerment, mental health

## Abstract

**Introduction:**

Recovery Colleges (RCs) are educational hubs offering free courses on mental health, well-being, and recovery through mutual and transformative learning. These co-learning spaces bring together individuals with diverse backgrounds—such as those with lived experience of mental illness, family members, and mental health practitioners—to collaboratively produce knowledge on mental health topics. Studies have shown RC participation leads to improvements in several psychosocial dimensions (e.g. mental health literacy, empowerment, well-being, reduced anxiety, stigma) and healthcare utilization. However, the methodological approach of averaging outcomes across all participants can mask important individual differences in experiences and outcomes, which is particularly significant given the heterogeneity of RC learners. In light of these limitations, this study aims to explore the heterogeneity of change among RC learners by identifying different trajectories of change and exploring their determinants.

**Methods:**

The study adopts a quasi-experimental longitudinal design with repeated measures, utilizing data from 353 participants recruited from a French-language RC in Quebec, Canada. Data were collected at three time points: baseline (T0) prior to program participation, one-month post-program (T1), and three to four months post-program (T2). The study uses clustering techniques to identify distinct patterns of change across participants, focusing on key outcome measures such as well-being, anxiety, resilience, empowerment, and stigma.

**Results:**

The results identified three distinct clusters of change trajectories. The largest cluster (Cluster A) demonstrated moderate improvements in well-being, anxiety reduction, and slight increases in empowerment and resilience. Cluster B, characterized by participants with higher baseline well-being and lower stigma, showed improvements in empowerment and a slight reduction in stigma, often linked to participants with clinical backgrounds, such as healthcare practitioners. Cluster C, primarily composed of participants with clinical levels of anxiety and lower baseline empowerment, exhibited significant reductions in anxiety and increases in empowerment over time.

**Discussion:**

This study contributes to a more nuanced understanding of the diverse outcomes associated with RC participation and highlights the importance of tailoring RC programs to meet the heterogeneous needs of learners. It also reinforces the role of empowerment as a central mechanism of change within the RC model, suggesting that empowerment fosters not only personal growth but also improved well-being and reduced stigma.

## 1 Introduction

Recovery Colleges (RCs) are educational hubs, based on the principles of mutual and transformative learning, providing free courses on mental health, well-being, recovery and living well collectively (1–4). Setting up an RC entails the development of a co-learning spaces opened to learners with different backgrounds (i.e. people living with mental illness, family members, practitioners and others) engage collaboratively in co-producing integrated knowledge within a transformative learning environment (3–5). A distinctive feature of the RC approach is its emphasis on the complementarity of different types of knowledge. This implies the participation of people with diverse and complementary backgrounds (experiential, clinical, theoretical) in a collaborative co-production process, in which each person’s contributions are equally recognized and valued (6, 7).

Several studies have reported the positive effects that participation in RCs can have for different types of participants using quantitative or qualitative design. Research findings have shown that attending a RC improves empowerment and self-worth (8–10), wellbeing (8, 10–13), personal growth and recovery (8, 9, 11, 13, 14), interpersonal skills and social connectedness (3, 8, 9, 14). Moreover, RC attendance may reduce self-reported anxiety symptoms (10, 12) and health care utilization (15–18). RC supports the empowerment of people living with mental illness and family members by an invitation to get involved, to open their horizons to new opportunities, to develop self-advocacy skills, to reclaim their right and agency over their lives (3, 8, 13, 14, 19). As a result of their participation in a RC course, mental health practitioners adhere more closely to recovery-oriented practices, have a renewed openness to others and to the value of experiential knowledge, develop reflective practice about their actions and new clinical skills, as well as re-engagement and commitment to their work (2, 3,10 20–22). For all learners, RC facilitates a change in attitudes towards mental health and reduces stigma (20, 23). Similar results were also replicated for the online format (10, 12, 29).

Despite the emphasis that RC framework poses on the importance of individual aspects of learning (25), aforementioned outcome studies were designed to provide an estimate of the average effect that participation in RC courses had on the “average” participant, as if the effects were the same for all participants and the participants had similar characteristics. From a methodological perspective, overall outcome analysis may mask differences across participants, are sensitive to outliers and may not represent adequately the target populations when it is very heterogeneous (26). Previous studies, both in the field of intervention evaluation in mental health (27, 28) and in the field of learning (29), have shown that different patterns of change can be observed within the studied sample, which can be summarized through statistical clustering techniques. We believe that not only can these issues be directly applied in RC outcome assessment as well, but that they are critical given the philosophical foundations and guiding principles of RC. In fact, RC is not an intervention intended for a uniform and specific target population: learners participating in RC have heterogeneous profiles as well as different motivations and purposes in participating in it. Thus, since RC learners may have different backgrounds and drives to attend RC, this heterogeneity will lead to different outcomes or patterns of change.

In view of these considerations, the aim of this study is to provide a better understanding of RC learners’ trajectories of change to better comprehend its heterogeneity. To achieve this aim, the specific objectives are: (1) to provide evidence of the heterogeneity of change across RC learners by clustering patterns of change; (2) to explore determinants of change patterns. Our interest is in being able to improve the effects of the model according to the specific needs of learners, which may differ from one learner profile to another. A better understanding of trajectories of change enables a better understanding of the target population in all its heterogeneity, and a better response to specific needs. Our interest is not in profiling, given that we’re in an inclusive model where individual distinctions are important and desirable.

## 2 Methods

### 2.1 Study design, participants’ recruitment and data collection

This pre-experimental study used a one-group pre-post test design with repeated measures. The baseline data collection (T0) took place prior to program participation during the registration process. The second data collection (T1) took place within one month after the end of the RC course program. The third data collection (T2) took place within three or four months after the end of the session.

Participants were recruited within the *Centre d’Apprentissage Santé et Rétablissement* (CASR), the sole French-language RC in Canada. In fall 2019, CASR started providing an RC curriculum and courses to Québec's general population. Since its inception, CASR ‘s has been developed with the idea of offering an inclusive experience open to the whole population, including people living with mental illness, family members, practitioners and other citizens. CASR was conceived as a public health initiative, particularly relevant during the COVID-19 pandemic, and as a space for collective learning addressing mental health literacy, self-management skills and stigma. In fall 2020, due to Covid-19 pandemic, CASR introduced a series of short online RC courses (three two-hour sessions totaling six hours per course) designed to enhance courses’ accessibility. All courses are co-designed and delivered by a pair of certified facilitators—one bringing experiential knowledge and the other offering clinical or theoretical expertise. Despite the differences in background and knowledge, the integration of different types of knowledge is valued. During the co-production process, facilitators use self-observation tools to ensure that the course aligns with the key principles of RC. Courses are offered in three annual sessions (spring, fall, and winter). In each session, a course catalog is made available, and learners may enroll in one course per session, with the opportunity to take additional courses across different sessions. Since fall 2020, CASR offered over 150 online courses lasting 6 hours (three 2-hour periods) to more than 3500 different learners.

Data collection was carried out through an online survey with Google Forms platform that collected baseline participants characteristics and outcome scales. The survey link was e-mailed to participants at T0, T1 and T2 (before the training course, after it, and 3 months later for a follow-up), and targeted reminders were sent to those who had not yet completed the survey.

Out of 419 participants, 66 were missing both T1 and T2 scores, categorizing them as study dropouts. These participants were excluded from the analysis. No statistically significant difference was found between final participants and dropouts.

### 2.2 Instruments

#### 2.2.1 Participants’ baseline characteristics

An *ad hoc* questionnaire was designed to collect participants’ characteristics. Items included relevant sociodemographic information, such as gender, age, country of birth, language spoken at home, level of education. Moreover, participants were asked to indicate how they would identify themselves, i.e. healthcare practitioner, administrative staff, person with lived experience or family member, college or university student, or “other”. They were also invited to select the types of knowledge they have on mental health, namely experiential, clinical or theoretical knowledge. Experiential knowledge is here defined as knowledge derived from direct knowledge of a mental health problem in private life. Clinical knowledge is knowledge developed through practice in a clinical setting, within a clinical role. Theoretical knowledge is gained through conceptual theoretical learning of mental health concepts. Finally, the questionnaire asked participants to indicate if they received, during their lifetime, a diagnosis of mental health condition and if they received any kind of mental health services in the last 6 months.

#### 2.2.2 Outcome measures

Validated questionnaires were chosen to evaluate outcome, operationalized in the different dimensions of well-being, anxiety, resilience, empowerment and stigma.

Wellbeing was measured using the Warwick-Edinburgh Mental Wellbeing Scale - Short Form (SWEMWS-7) (30). The SWEMWS-7 is a 7-item measure that uses a five-point Likert scale to identify mental wellbeing and overall satisfaction with life.

The Generalized Anxiety Disorder-7 questionnaire (GAD-7) (31) is a seven-item scale used to assess the frequency of anxiety symptoms experienced over the past two weeks, serving as an indicator of psychological distress. It has been widely employed in population-based research (32) as well as in clinical outcome studies. Each item is rated on a scale from 0 to 4, with total scores above 8 indicating a clinically significant level of anxiety

The Connor-Davidson Resilience Scale (CD-RISC-10) (33) assessed resilience using a 10-item measure based on a five-point Likert scale. Since it demonstrates a unidimensional structure, the total score (α = 0.85) was adopted in the study.

Empowerment was measured using the Consumer Constructed Scale to Measure Empowerment (CCSME) (34). The CCSME is a 25-item measure that uses a four-point ordinal scale to measure personal and community empowerment of people living with mental illness (35). The total scale score, whose internal consistency is 0.84, was used in the present study.

Stigma was measured using the Opening Minds Scale for Health Care Providers (OMS-HC) (36). The OMS is a 15-item measure that uses a five-point Likert scale to identify stigmatizing attitudes towards people with mental health problems, openness to disclosure and help-seeking and social distance.

### 2.3 Data analysis

#### 2.3.1 Dataset preparation and sample description

For handling missing values in outcome scales, cases with missing data at T1 or T2 were retained, and the missing values were imputed using a linear model. This model estimated the missing values based on independent variables, including each scale score at T0 and participants’ characteristics. Additionally, three cases were removed due to missing predictors. The total sample used for analysis consisted of 353 valid participants, whose descriptive statistics are presented in the results paragraph. Moreover, 39 participants attended more than one course at different sessions (e.g. in spring 2021, then in fall 2021), resulting in their recurrence in multiple rows of the dataset. Therefore, the full dataset represents 393 data rows, each one corresponding to individual outcome scores a T0, T1 and T2 for a single session. Overall outcome was assessed, for each variable, comparing mean scores using t-test and computing effect size (Hedge’s G formula). From now on, we will call individual trajectory the combination of scores at T0, T1, T2 of an individual participant in a specific session.

#### 2.3.2 Cluster analysis

Cluster analysis was conducted on 393 trajectories. To ensure a more rigorous approach to cluster analyses, we conducted these analyses with three methods, i.e. joint trajectory k-means (KML3D), standard k-means algorithm (KM) and hierarchical clustering (HCLUS). Raw scales scores were standardized before the clustering procedure. Moreover, for KM and HCLUS, principal component analysis was performed to reduce the number of dimensions. For each algorithm, the best number of clusters was determined using functions internal to the algorithm, namely: Calinsky-Harabatz for KML3D, and the elbow method for KM and HCLUS. Therefore, each change trajectory was classified three times independently by three different classification algorithms; then, the final cluster was assigned by applying the majority vote method (37), which involves choosing the cluster most frequently attributed by each of the different algorithms.

For each cluster, descriptive statistics and outcome assessment was performed. Predictors of cluster membership (cluster B and C *vs* A) were assessed running a multinomial logistic regression model using VGLM package in R, entering participants’ baseline characteristics as predictors. However, outcome scales scores at T0 were not included in the model, since this information was already used in clustering algorithm. The odds ratio (OR) and 95% confidence limits (CL) were computed for each predictor. Low to moderate levels of collinearity were assessed and evaluated acceptable for the analysis.

## 3 Results

### 3.1 Sample description

Sample description is presented in **Table 1**. The age distribution of participants is evenly concentrated between the ages of 18 and 49, with a smaller percentage aged 50 or over. The majority were female (85.6%), with males making up 13.3%. Healthcare and educational practitioners formed the largest group of learners (41.36%), followed by persons with lived experience or family members (17%) and manager/administrative staff (16.43%). Most participants had experiential (71.95%) and theoretical (71.67%) mental health knowledge, with a significant portion having clinical knowledge (55.81%). Nearly half of the participants (49.01%) had received a mental illness diagnosis in their lifetime, and 40.51% had received mental health services in the last six months. The majority attended the course for the first time (89.82%).

**Table 1:** Sample description at baseline (N = 353).

Age	
18 – 29	82 (23.23%)
30 – 39	77 (21.81%)
40 – 49	84 (23.8%)
50 – 59	62 (17.56%)
60+	45 (12.75%)
Biological gender, male	
Female	302 (85.6%)
Male	47 (13.3%)
Non binary/other	4 (1.1%)
Type of learner	
Healthcare and educational practitioner	146 (41.36%)
Person with lived experience or family member	60 (17%)
Manager and administrative staff	58 (16.43%)
College or university student	37 (10.48%)
Other kind of citizen	48 (13.6%)
Mental health knowledge	
Experiential	254 (71.95%)
Theoretical	253 (71.67%)
Clinical	197 (55.81%)
Mental health parameters	
Received a diagnosis of mental illness lifetime	173 (49.01%)
Received mental health services in last 6 months	143 (40.51%)
Turn/time of course attended	
First time	353 (89.82%)
Second or more time	40 (10.18%)
First session attended	
Fall 2020	49 (12.47%)
Spring 2021	63 (16.03%)
Fall 2021	50 (12.72%)
Winter 2022	57 (14.5%)
Spring 2022	37 (9.41%)
Fall 2022	55 (13.99%)
Winter 2023	63 (16.03%)
Spring 2023	19 (4.83%)

### 3.2 Overall outcome

**Figure 1:**
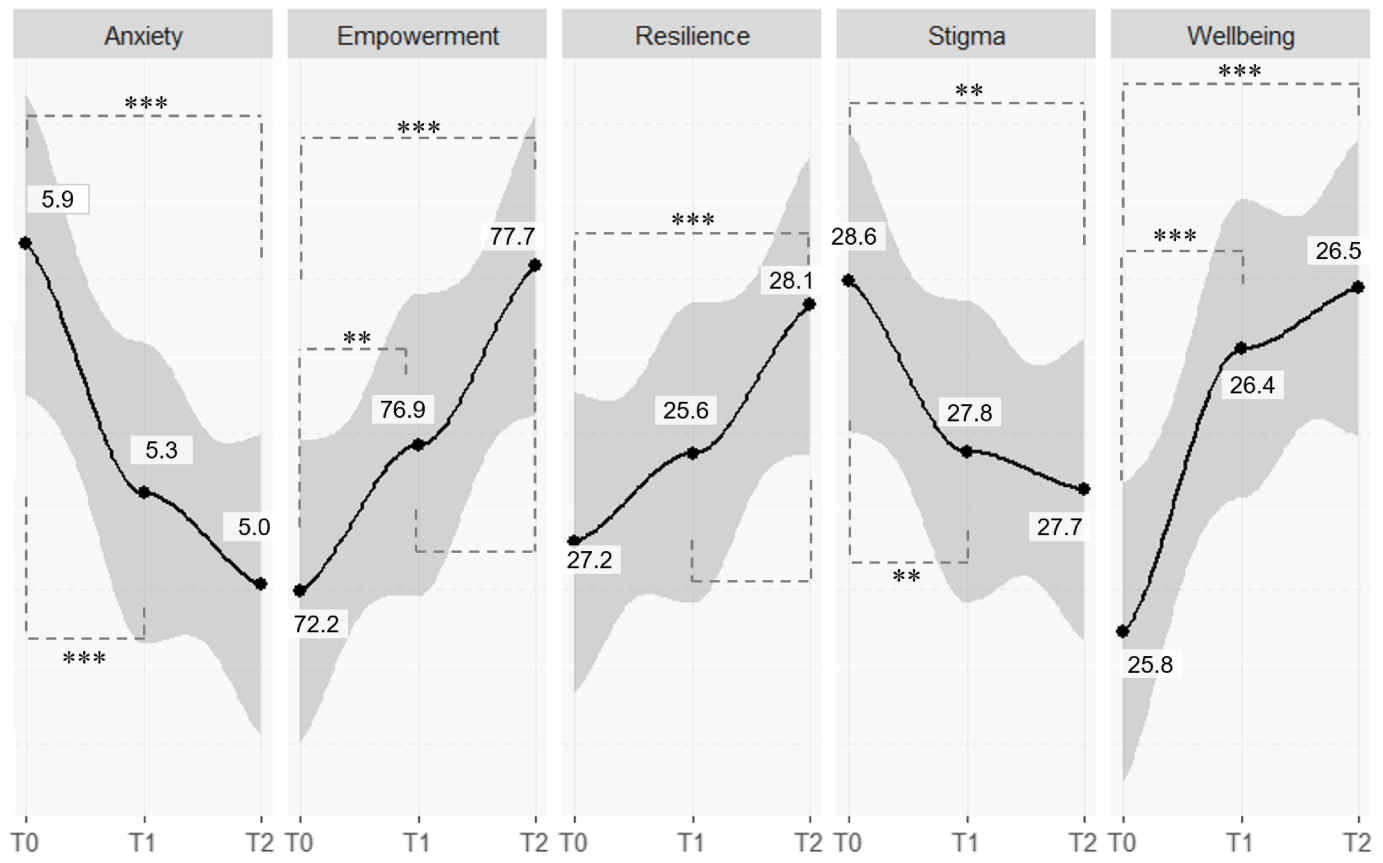
Overall outcome evaluation, average trajectories and statistically significant changes across time. ** = p < 0.01; *** = p < 0.001.

**Table 2** and **Figure 1** shows changes in outcome measures across T0, T1 and T2. Wellbeing increased significantly from 25.8 (SD = 3.4) at T0 to 26.4 (SD = 3.3) at T1 and maintained the same level (mean 26.5, SD = 3.5) at T2. Anxiety decreased significantly from 5.9 (SD = 4.2) at T0 to 5.3 (SD = 3.9) at T1 and decreased to a lesser extent (mean 5.0, SD = 4.1) at T2. Empowerment demonstrated constant statistically significant improvement, rising from 72.2 (SD = 6.6) at T0 to 76.9 (SD = 6.9) at T1 and 77.7 (SD = 6.8) at T2. Resilience slightly improved from T0 to (mean 27.2, SD = 5.9) to T1 (mean 27.6, SD = 5.8) and from T1 to T2 (mean 28.1, SD = 5.8), and change was statistically significant between T1 and T2 and between T0 and T2. Stigma showed a slight decline, decreasing significantly from 28.6 (SD = 6.8) at T0 to 27.8 (SD = 6.3) at T1, and then maintain this level even at T2 (mean 27.7, SD = 6.2).

**Table 2:** Overall outcome scores, 393 individual trajectories.

Outcome measures	T0	T1	T2	T1-T0		T2-T1		T2-T0	
	Mean (SD)	Mean (SD)	Mean (SD)	ES	t value	ES	t value	ES	t value
Wellbeing	25.8 (3.4)	26.4 (3.3)	26.5 (3.5)	0.19	4 ***	0.04	0.8 ns	0.22	4 ***
Anxiety	5.9 (4.2)	5.3 (3.9)	5.0 (4.1)	-0.16	-4 ***	-0.06	-1 ns	-0.22	-5 ***
Empowerment	72.2 (6.6)	76.9 (6.9)	77.7 (6.8)	0.10	3 **	0.12	3 **	0.22	5 ***
Resilience	27.2 (5.9)	27.6 (5.8)	28.1 (5.8)	0.06	2 ns	0.10	2 *	0.15	4 ***
Stigma	28.6 (6.8)	27.8 (6.3)	27.7 (6.2)	-0.11	-3 **	-0.03	-0.7 ns	-0.13	-3 **

ES, effect size; * = p < 0.05; ** = p < 0.01; *** = p < 0.001; ns, not statistically significant (p ≥ 0.05).

### 3.3 Identification and description of clusters

Joint application of the three cluster analysis algorithms allowed the trajectories of change to be grouped into three clusters. The consensus among the three methods was complete (i.e., the same cluster was attributed by all three algorithms independently) for 387 out of 393 individual trajectories, while it was partial (i.e., the same cluster was attributed 2 out of 3 algorithms) for 6 trajectories.

**Figure 2:**
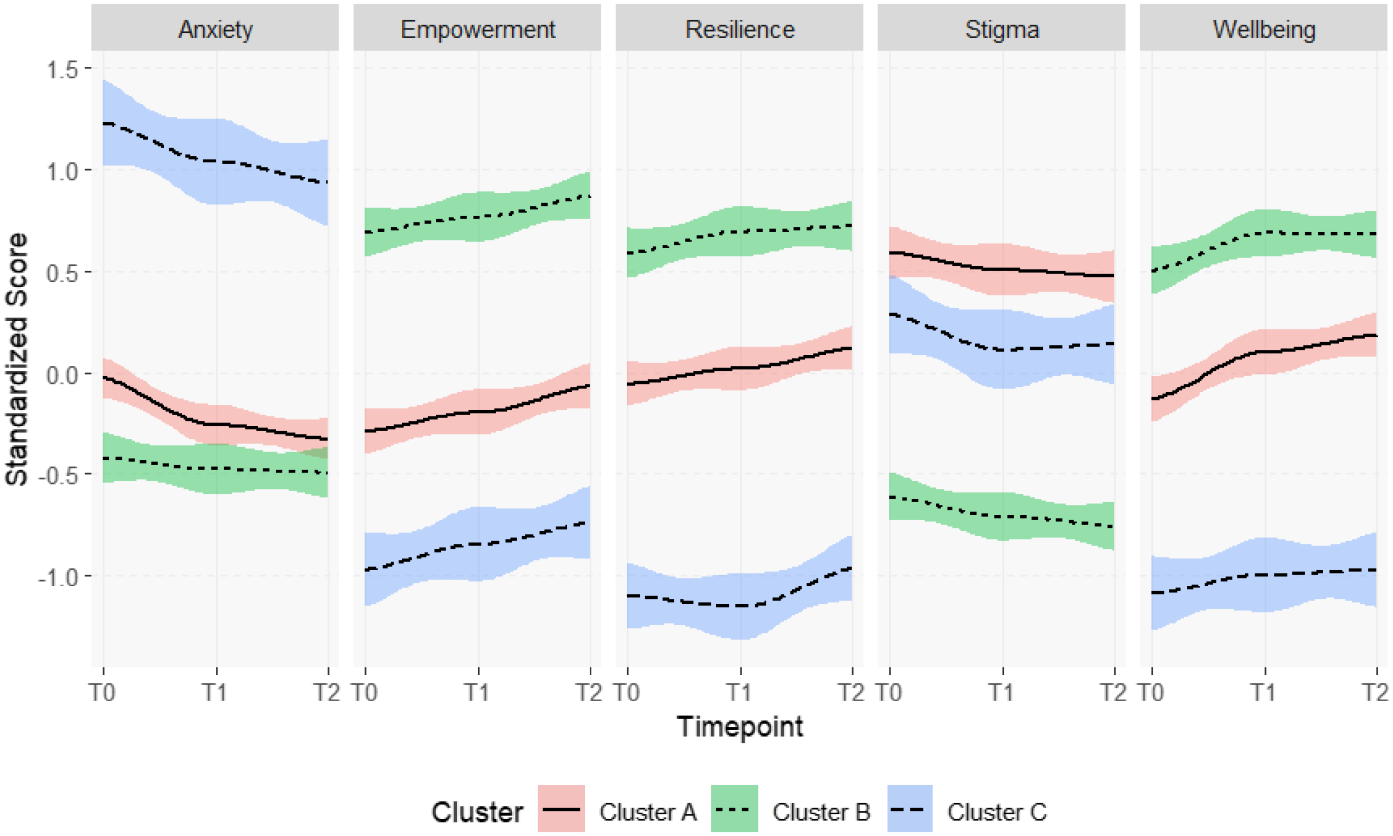
Comparison of trajectories of change for each outcome variable between clusters. Coloured areas represent change trajectories for each cluster. Study: Clustering change patterns among learners of an online Recovery College in Quebec, Canada.

**Table 3** and **Figure 2** present the outcome measures scores for three clusters of individual trajectories at three different time points (T0, T1, T2). The table provides descriptive statistics, and an assessment of changes that occurred between these time points, including t-test and effect sizes. **Figure 2** plots standardized mean scores with coincidence intervals for each outcome variable, to provide a graphical representation of the average trajectory for each cluster. Cluster A was the larger cluster, grouping together 165 (42.0%) trajectories. Within this cluster, the mean wellbeing scores significantly increased between T0 and T1 (t=-3, p<0.001) and change was statistically significant also at T2, with an overall effect size of 0.43. Anxiety scores showed a similar pattern, with statistically significant reduction between T0 and T1 (t=-5, p<0.001) that lasted also at T2, with an overall effect size of -0.44 (t=-5, p<0.001). For empowerment and resilience, scores slighted increased at T1 and T2, but only the difference between T0 and T2 was statistically significant (CCSME: t=3, p<0.01; CD-RISC10: t=3, p<0.001) with an effect size of 0.31. Stigma scores slightly decreased at T1 and T2, but changes were statistically not significant.

**Table 3:** Change in mean score over time for each cluster of individual trajectories.

Outcome	T0	T1	T2	T1-T0		p	T2-T1		p	T2-T0		p
	Mean (SD)	Mean (SD)	Mean (SD)	*ES*	t value		*ES*	t value		*ES*	t value	
Cluster A (N = 165)												
Wellbeing	25.7 (2.7)	26.6 (2.5)	26.9 (2.4)	*0.31*	-3	***	*0.12*	1		*0.43*	4	***
Anxiety	5.3 (3.0)	4.3 (2.5)	4.1 (2.6)	*-0.35*	-5	***	*-0.11*	-1		*-0.44*	-5	***
Empowerment	74.9 (4.7)	75.6 (5.1)	76.5 (5.2)	*0.13*	2		*0.17*	2		*0.31*	3	**
Resilience	27.3 (4.7)	27.8 (3.9)	28.4 (3.9)	*0.10*	1		*0.15*	2		*0.25*	3	***
Stigma	31.8 (6.5)	31.2 (5.5)	31.0 (5.4)	*-0.10*	-1		*-0.04*	-0.6		*-0.14*	-2	
Cluster B (N = 138)												
Wellbeing	28.0 (2.5)	28.6 (2.1)	28.6 (2.7)	*0.28*	3	**	*0.01*	-0.07		*0.25*	2	*
Anxiety	3.7 (2.9)	3.4 (2.9)	3.4 (3.4)	*-0.08*	-1		*-0.02*	-0.3		*-0.10*	-1	
Empowerment	81.7 (4.5)	82.2 (5.1)	83.0 (5.3)	*0.10*	1		*0.14*	2		*0.25*	3	**
Resilience	31.2 (4.3)	31.8 (4.2)	32.0 (4.7)	*0.15*	2		*0.04*	0.4		*0.18*	2	
Stigma	23.9 (4.9)	23.3 (4.3)	22.9 (4. 6)	*-0.14*	-2		*-0.07*	-0.8		*-0.20*	-2	*
Cluster C (N = 90)												
Wellbeing	22.4 (2.9)	22.7 (2.8)	22.8 (3.5)	*0.11*	1		*0.03*	0.03		*0.13*	1	
Anxiety	10.5 (4.3)	9.7 (4.1)	9.3 (4.4)	*-0.19*	-2		*-0.10*	-0.8		*-0.28*	-2	*
Empowerment	70.2 (6.0)	71.1 (6.5)	71.8 (5.7)	*0.14*	2		*0.1*	1		*0.27*	3	**
Resilience	21.1 (4.8)	20.8 (4.1)	21.9 (5.1)	*- 0.07*	0.7		*-0.25*	-2	*	*0.17*	1	
Stigma	29.8 (6.8)	28.6 (6.5)	28.8 (5.2)	*-0.17*	-2	*	*0.03*	0.3		*-0.16*	-2	

ES, effect size; * = p < 0.05; ** = p < 0.01; *** = p < 0.001.

One hundred thirty-eight (33.11%) trajectories were grouped in cluster B. In this cluster, wellbeing scores significantly increased from T0 to T1 (t=3, p<0.01), and changes were maintained at T2 (t=2, p<0.05) with an effect size of 0.25. Empowerment scores progressively increased at T1 and T2, and difference between T2 and T0 was statistically significant (t=3, p<0.01) with an effect size of 0.25. A slight but progressive decrease was also observed for stigma scores, and comparison betweenT0 and T2 was statistically significant (t=-2, p<0.05) with an effect size of -0.20. No statistically significant changes were found in anxiety and resilience in this cluster.

In cluster C, 90 (22%) trajectories were grouped together. In this cluster, slight but progressive changes were observed at T1 and T2 in in anxiety (decrease) and empowerment (increase), which were significant when comparing T0 and T2 (GAD7_T2-T0_: t=-2, p<0.05, e.s. = -0.28; CCSME_T2-T0_: t=3, p<0.01, e.s. = 0.27). For stigma, a significant decrease was observed between T0 and T1 (t=-2, p<0.05) which, however, was not maintained at T2, where the comparison with T0 was nonsignificant. Regarding resilience, a slight significant change was observed in the comparison between T2 and T1 (t=-2, p<0.05), but the comparison with T0 was not significant. Wellbeing scores slightly increased at T1 and T2, but changes were statistically not significant.

### 3.4 Determinants of change trajectories

**Table 4** presents results of the multinomial logistic regression. Types of mental health knowledge variables were significant predictors: in fact, having a clinical knowledge increased the odds of cluster B compared to Cluster A (OR = 2.25, p < 0.01), whereas having a theoretical knowledge decreased the odds of Cluster C (OR = 0.32, p < 0.01). Moreover, having received a diagnosis of mental illness in a lifetime (OR = 2.93, p < 0.01) or mental health services in the last six months (OR = 3.79, p < 0.001) were significant predictors for Cluster C, compared to cluster A.

**Table 4:** Sociodemographic characteristics associated with cluster membership.

Determinants	Cluster A	Cluster B *vs* Cluster A		Cluster C *vs* Cluster A	
	Freq. (%)	Freq. (%)	OR (95% CI)	Freq. (%)	OR (95% CI)
**Intercept**	n.a.	n.a.	0.58 (0.19-1.74)	n.a.	0.34 (0.08-1.41)
Age ^a^					
18 - 29	43 (26.06%)	25 (18.12%)	0.71 (0.31-1.63)	18 (20%)	1.28 (0.48-3.41)
30 - 39	28 (16.97%)	37 (26.81%)	1.52 (0.72-3.23)	19 (21.11%)	1.9 (0.74-4.88)
40 - 49	38 (23.03%)	30 (21.74%)	Ref. cat.	20 (22.22%)	Ref. cat.
50 - 59	28 (16.97%)	30 (21.74%)	1.54 (0.71-3.37)	15 (16.67%)	0.48 (0.18-1.33)
60+	26 (15.76%)	16 (11.59%)	0.95 (0.39-2.28)	17 (18.89%)	0.4 (0.14-1.14)
Biological gender					
Female	138 (83.6%)	119 (86.2%)	Ref. cat.	80 (88.9%)	Ref. cat.
Male and non-binary^b^	27 (16.4%)	19 (13.8%)	0.52 (0.22-1.25)	10 (11.1%)	1.28 (0.48-3.41)
Type of learner ^a^					
Healthcare and educational practitioner	62 (37.58%)	73 (52.9%)	Ref. cat.	24 (26.67%)	Ref. cat.
Manager and administrative staff	29 (17.58%)	19 (13.77%)	0.78 (0.34-1.81)	13 (14.44%)	1.14 (0.39-3.35)
Person with lived experience or family member	26 (15.76%)	20 (14.49%)	0.74 (0.31-1.75)	24 (26.67%)	2.49 (0.89-6.99)
College or university student	19 (11.52%)	12 (8.7%)	1.39 (0.5-3.86)	7 (7.78%)	1.05 (0.29-3.78)
Other kind of citizen	26 (15.76%)	14 (10.14%)	0.65 (0.27-1.57)	21 (23.33%)	1.57 (0.58-4.26)
Mental health knowledge					
Experiential ^c^	114 (69.09%)	100 (72.46%)	1.27 (0.68-2.37)	76 (84.44%)	0.71 (0.29-1.74)
Theoretical ^c^	121 (73.33%)	105 (76.09%)	0.75 (0.4-1.4)	48 (53.33%)	0.32 (0.15-0.65) **
Clinical ^c^	83 (50.3%)	96 (69.57%)	2.24 (1.23-4.06) **	38 (42.22%)	0.88 (0.44-1.78)
Mental health parameters					
Received a diagnosis of mental illness lifetime ^c^	73 (44.24%)	67 (48.55%)	1.09 (0.61-1.97)	67 (74.44%)	2.93 (1.38-6.23) **
Received mental health services in last 6 months ^c^	52 (31.52%)	53 (38.41%)	1.23 (0.71-2.11)	63 (70%)	3.79 (1.98-7.26) ***
Turn/time of course attended					
First time	152 (92.12%)	127 (92.03%)	Ref. cat.	74 (82.22%)	Ref. cat.
Second or more time	13 (7.88%)	11 (7.97%)	0.82 (0.33-2.04)	16 (17.78%)	1.5 (0.59-3.81)
First session attended ^a^					
Fall 2020	24 (14.55%)	11 (7.97%)	0.64 (0.25-1.66)	14 (15.56%)	1 (0.32-3.12)
Spring 2021	27 (16.36%)	25 (18.12%)	Ref. cat.	11 (12.22%)	Ref. cat.
Fall 2021	25 (15.15%)	16 (11.59%)	0.71 (0.3-1.69)	9 (10%)	0.94 (0.3-2.9)
Winter 2022	23 (13.94%)	24 (17.39%)	1.13 (0.49-2.61)	10 (11.11%)	0.72 (0.23-2.31)
Spring 2022	14 (8.48%)	15 (10.87%)	1.08 (0.41-2.82)	8 (8.89%)	0.88 (0.26-3.02)
Fall 2022	19 (11.52%)	18 (13.04%)	0.97 (0.4-2.34)	18 (20%)	1.91 (0.64-5.71)
Winter 2023	26 (15.76%)	20 (14.49%)	0.78 (0.34-1.81)	17 (18.89%)	1.03 (0.36-2.93)
Spring 2023	7 (4.24%)	9 (6.52%)	1.29 (0.39-4.24)	3 (3.33%)	1.13 (0.22-5.94)

^a^ = comparison made simultaneously on all levels of the nonbinary variable; reference cat. = reference category, chosen as the category with higher frequency;

^b^ = non-binary (n = 4); ^c^ =binary variable, reference is 0 (= absence of the characteristic); ** = p < 0.01; *** = p < 0.001.

Multinomial logistic regression was used to assess the predictive value of each sociodemographic variable for cluster membership, using Cluster A as the reference.

## 4 Discussion

The objectives of the present study were to provide evidence of the heterogeneity of change across RC learners by clustering patterns of change and to explore determinants of change patterns. To accomplish the first objective, a cluster analytic strategy was applied to outcome scales total score at T0, T1 and T2, and a three-cluster solution was adopted.

Cluster A, the largest subgroup, represented four out of ten change trajectories. The pattern of change for cluster A could be described as a moderate improvement in wellbeing, moderate reduction in anxiety and slight but significant increases in empowerment and resilience. This cluster shows the most consistent effect sizes (>.40), particularly for increases in well-being and reductions in anxiety. These effects are more pronounced than those observed in the total sample and in the subsequent clusters, suggesting that this group benefits the most from participation. This type of trajectory appears to be the most typical in participants and does not seem to be associated with any specific sociodemographic characteristics. The Cluster A trajectory aligns with previous findings from RC studies (2, 10) that documented enhancement in wellbeing, empowerment and resilience. These consistent outcomes suggest that the moderate but significant changes observed in Cluster A are representative of the typical benefits gained through participation in Recovery Colleges that are already presented in previous outcome studies. At the same time, the trajectories observed for Clusters B and C are more specific and particular and may provide insights for conceptualization and planning of future RCs.

Cluster B trajectories, that were most frequently associated with clinical knowledge, have distinctive elements from the previous one. First, slight increases are observed in wellbeing (which has the highest values at T0) and empowerment. Secondly, this cluster depicted a slight but significant decrease in stigma levels. Overall, we may speculate that this type of trajectory was more typical for health or education partitioners who want to develop new perspectives on mental health by directly engaging in an experiential learning process. Previous findings of Briand and colleagues (10), who analyzed an earlier version of this database using OMS-HCP subscales scores, showed that the most pronounced change at T2 was an increased willingness to seek help or to self-disclose. Research indicates that healthcare practitioners often face higher distress levels but are reluctant to disclose mental illness or seek treatment due to stigma (38–41). Moreover, reluctance toward help-seeking behaviors increases as practitioners gain experience, despite the need for psychological support (38). We suggest that the distinctive features of the online RC college facilitated a change in their attitudes toward help-seeking and thus improved their well-being. These results echo the ones from previous studies (20, 21, 42) that found that Recovery Colleges positively impact on the mental health of practitioners by modifying their attitudes and their practices, leading to increased work motivation. The decrease in stigma levels observed in Cluster B may reflect similar changes in attitudes among health or education practitioners participating in Recovery Colleges.

In Cluster C, the typical trajectory involved a decrease in anxiety and an increase in empowerment without improvements in well-being and resilience as well as in Cluster A. Interestingly, this trajectory is more frequent for people who have received a diagnosis of mental illness in their lifetimes or who have received mental health services in the previous six months. These results align with previous studies indicating that Recovery Colleges offer a supportive environment that facilitates the recovery process for individuals living with mental health conditions (3). The increase in empowerment and decrease in anxiety observed in Cluster C may be attributed to the RC’s role in helping individuals shift from a patient identity to a student identity, thereby reducing stigma and fostering hope (43). Additionally, improvements in self-esteem, confidence, and interpersonal skills reported by participants in other studies (8) support the notion that RC participation can lead to significant personal growth for those with clinical mental health conditions.

The overall results allow us to introduce several more general level reflections on the effect of participation in RC courses. First, that patterns of change may be partially associated to participant’s characteristics; however, although there are associations between some characteristics (e.g., clinical knowledge or having a mental health problem) and some trajectories, this association is partial and probabilistic. Second, the greatest benefits—in terms of increased well-being and reduced anxiety—are observed in approximately half of the participants (Cluster A). In contrast, the remaining participants, more frequently comprising healthcare practitioners (Cluster B) or individuals with more recent or direct experience of mental health problems (Cluster C), exhibit more modest changes, with small effect sizes. However, we believe that the interpretation of effect sizes should be contextualized by considering that RC participants did not receive a clinical intervention, but rather took part in a brief online course consisting of just three sessions—a low-intensity learning experience that nonetheless produced measurable change. Third, results suggest that increase in empowerment seems to be the most common outcome occurring in all three clusters. This finding is coherent with RC approach in which an empowering environment, where personal choices are supported, safety and respect are promoted and recovery-oriented processes are embodied, is considered as one of the main mechanisms of change (5). The RC change model hypothesizes that the RC course environment provides the context for a learner’s empowering experience that involves the creation of a relational environment where the interactions with trainers (both clinicians and experts by experience) and other learners are different from those usually experienced in traditional clinical settings (5, 24). By examining power relations, establishing more egalitarian relationships and co-producing knowledge, learners can develop new relationships, enhance their critical awareness and sense of self-efficacy, as well as exert more influence. Acquiring new knowledge, self-management strategies combined with experiencing a more active role in the RC context lays the foundation for increasing the sense of agency, defined as the ability to express one’s goals and act on them (44). Setting up such an innovative and nurturing environment promotes a new perspective on mental health (3, 30). This facilitates the development of more humane and reciprocal relationships, especially between health practitioners (or learners with clinical knowledges) and people with lived experience (1, 5). This mechanism of change is in line with Zimmerman and Warschausky’s concept of psychological empowerment (45), which combines intrapersonal, interactional and behavioral components to the construct. Future research could provide a more detailed analysis of the relationship between the enabling learning environment and empowerment, examining empowerment both as a dynamic process and as an outcome.

### 4.1 Strengths and limitations

The present study has strengths and limitations that need to be considered. The strongest asset of this study is the sample size. The study assesses pre- and post-intervention changes in the psychosocial dimension across a diverse sample of 343 participants, which is notably larger than most studies that have previously evaluated the RC outcome using self-administered scales (46, 47), excluding previous articles published on earlier version of the same dataset (10, 12).

In addition, another relevant aspect is the composition of the sample, which is not only based on mental health service users but includes a variety of social roles and knowledge (experiential, clinical, theoretical). This element is consistent with the original vision of RC, understood as an intervention for all citizens and valuing the diversity of knowledge.

At the same time, the study has some methodological limitations that must be considered in order to properly evaluate the results obtained. The first limitation is the adoption of a pre-post design without a control group, which limits the ability to draw causal inferences about the effects of RC participation. This choice was informed by the nature of the study, which aimed to evaluate the outcomes of an existing RC program attended by self-selected citizens. Implementing a control group within this context posed practical and ethical challenges. However, future research could address this limitation by exploring alternative designs—such as the use of waiting list controls or comparison groups attending non-RC-based mental health courses—to strengthen causal interpretations. Another limitation is the reliance on self-reported measures. Self-reporting can introduce biases such as social desirability bias, recall bias, and subjective interpretation, which can affect the accuracy and reliability of the data collected. Participants might overestimate or underestimate their behaviors, attitudes, or experiences, leading to data that do not accurately reflect the true nature of the phenomena being studied. This can compromise the validity of the research findings and make it difficult to draw definitive conclusions.

Another limitation is the methodological choice to not use subscales for the outcome measures, but only total scores. Outcome measures often encompass multiple dimensions, and using subscales can provide a more nuanced understanding of the different aspects of the outcomes. By not employing subscales, the study may overlook critical variations within the data, leading to a less comprehensive analysis. This can result in a loss of valuable information that could inform more targeted interventions or further research. However, the decision to rely on total scores rather than subscales was made after careful consideration, particularly given the potential of subscales to capture more nuanced trends. However, three key observations led to the exclusion of subscales from the primary analyses: *a*) for most instruments, subscales were moderately correlated, meaning that overall trends captured by total scores would likely reflect similar patterns; *b*) statistical algorithms treat subscale scores as independent, and including instruments like the CCSME and OMS-HCP —both of which comprise multiple, intercorrelated subscales—could bias the analysis and disadvantage single-score tools such as the SWEMWS-7 and GAD-7; and *c*) the interpretation of clusters generated from a large number of subscales becomes cognitively demanding and less informative. Nonetheless, future research could delve more deeply into subscale-specific trends by employing fewer instruments and focusing on more targeted constructs, such as empowerment or stigma.

The choice of the number of clusters in the study also presents a significant limitation. There is always a level of arbitrariness in determining the number of clusters, which can affect the study’s outcomes. The clusters represent a synthesis of outcomes, and the selection process may be influenced by subjective judgment or methodological constraints rather than purely data-driven criteria. This arbitrariness can lead to clusters that may not accurately represent the underlying patterns in the data, thereby impacting the study’s validity and generalizability. Moreover, while clustering is a powerful tool for identifying patterns across multidimensional data, its inherent tendency to reduce complex information into simplified groupings can present significant limitations—particularly in the context of Recovery Colleges. These programs are grounded in values of personal meaning-making, agency, and the non-linear nature of recovery journeys (1). By collapsing diverse experiences into broad profiles, clustering may inadvertently obscure important variations in how individuals engage with and benefit from recovery-oriented learning environments. We recognize this as an important issue inherent in using clustering: the article aims to avoid the risk of reducing everything to a single outcome, yet still predicts a finite number (three). While the number of clusters could (at least theoretically) be increased, it would remain a simplification of the complexity and uniqueness of the people involved. To address this limitation, future research may integrate clustering with subgroup-focused analyses that attend to learner characteristics such as age, prior service use, or cultural background. These variables may shape recovery in ways that are not captured by outcome scores alone. Furthermore, integrating narrative or qualitative data can help preserve the richness of individual experience and ensure that statistical patterns are not misinterpreted as uniform trajectories. In doing so, research stays true to the ethos of Recovery Colleges and enhances the potential for meaningful, person-centered insights.

A further limitation concerns the validity and reliability of the items used to assess participants’ knowledge. Each item was accompanied by the definitions provided above; however, these definitions alone may not have been sufficient to offer a clear cognitive anchor, potentially limiting participants’ ability to accurately and consistently categorize their own knowledge. For instance, participants may have overestimated their theoretical knowledge. Moreover, the experiences that constitute experiential knowledge can vary widely—for example, knowing a family member with mild symptoms differs significantly from having lived experience of a severe mental disorder. Future research would benefit from the development of a validated scale to more precisely measure and classify mental health-related knowledge.

## 5 Conclusion

This study is the first attempt to describe possible different trajectories of change of RC learners. Findings highlight the heterogeneity of change trajectories among RC learners, emphasizing the complexity of individual growth within recovery college programs.

The results, in addition to confirming what is already known with respect to the positive effects of participation in Recovery College, invite reflection on two additional subgroups suggested by the trajectory analysis. The first concerns learners with a clinical background, for whom a combination of reduced stigma and increased well-being and empowerment is observed. On the policy level, this suggests how RC can be promoted as a useful intervention to support not only staff training but also practitioner well-being. The second concerns learners with more complex mental health issues for whom the benefits of participation in recovery college, while present, are more limited. This suggests the need to design RC course offerings specifically to meet the needs of these people. For example, courses with a longer duration or promoting more intensive participation could be offered.

Moreover, despite observed differences between clusters, a learning environment that clearly support psychological empowerment emerged as a central dimension of outcome. Future research should further explore how diverse profiles of learners engage with the RC environment and the extent to which empowerment processes mediate other positive outcomes. These insights can guide the tailoring of RC programs to better meet the varied needs of participants.

## Data Availability

The raw data supporting the conclusions of this article will be made available by the authors, without undue reservation.
